# Fibrous Dysplasia: An Overview of Disease Process, Indications for Surgical Management, and a Case Report

**Published:** 2015-02-26

**Authors:** Oluwaseun A. Adetayo, Samuel E. Salcedo, Vedant Borad, Sara S. Richards, Adrienne D. Workman, Andrea O. Ray

**Affiliations:** ^a^Division of Plastic Surgery, Albany Medical Center, Albany, NY; ^b^Department of Plastic Surgery, Loma Linda University Medical Center, Loma Linda, CA; ^c^Albany Medical College, Albany, NY

**Keywords:** fibrous dysplasia, craniofacial fibrous dysplasia, craniofacial reconstruction, PEEK implant, von Recklinghausen

## Abstract

**Introduction:** First described by Von Recklinghausen in 1891, fibrous dysplasia is a developmental defect of osseous tissue such that bone is produced with an abnormally thin cortex and marrow is replaced with fibrous tissue that demonstrates characteristic ground-glass appearance on x-ray examination. The underlying defect in fibrous dysplasia is a mutation of the *GNAS1* gene, which leads to constitutive activation of gene products that preclude the maturation of osteoprogenitor cells and lead to development of abnormal bone matrix, trabeculae, and collagen, produced by undifferentiated mesenchymal cells. There exists a mainly self-limiting form of fibrous dysplasia classified as monostotic, which is characterized by dysplastic bone in a single location that remains relatively stable throughout life and a polyostotic form, which can exhibit aggressive growth placing adjacent structures at risk for compressive sequelae. **Methods:** We present the surgical management of an unusual case of monostotic fibrous dysplasia, which exhibited aggressive growth with mass effect, and late presentation, both uncharacteristic features for the monostotic form. The authors also performed a comprehensive review of the literature and discuss the disease process, management options, and indications for surgical treatment. **Results:** An overview of the disease process and management options is presented. The authors also present details of reconstruction in an unusual form of symptomatic monostotic fibrous dysplasia. **Conclusion:** Conservative management is usually the mainstay of therapy in asymptomatic cases of fibrous dysplasia. In patients fulfilling criteria for surgical management, craniofacial reconstruction offers a viable option in the surgeon's armamentarium, providing good functional and cosmetic outcomes.

Fibrous dysplasia (FD) is a congenital skeletal disorder characterized by thinning of the cortex and replacement of the marrow with fibrous tissue, which can sometimes result in deformity, pain, pathologic fractures, loss of mechanical strength, and nerve entrapment.^1^^,^^2^ The disease, which was first reported by Von Recklinghausen in 1891,^3^^,^^4^ was later described by Lichtenstein in 1938^1^^,^^5^ and subsequently in 1942 along with Jaffe.^1^^,^^6^ The lesions have been reported to account for 2.5% to 7.0% of all benign bone tumors, with an equal predilection for both sexes.^1^^,^^3^ The underlying genetic abnormality is a postzygotic mutation of the *GNAS1* gene, resulting in a nonhereditary somatic mosaic state.^7^ The phenotypic expression in FD is directly related to the severity, clinical presentation, and subtype of the disease. The *GNAS1* gene is located at chromosome 20q13.2-13.3 and encodes the alpha subunit of the stimulatory G protein (G_s_α), which activates adenylyl cyclase following exchanging of guanosine triphosphate for guanosine diphosphate.^1^ This results in a gain-of-function mutation of the *GNAS1*, the result of which is loss of guanosine triphosphatase activity of the G_s_α subunit resulting in decreased catalysis of guanosine triphosphate to guanosine diphosphate. As a result, the mutated G_s_α leads to constitutive activation of adenylyl cyclase, leading to excess cyclic adenosine monophosphate.^7^^,^^8^ In bone, this upregulatory effect precludes the maturation of osteoprogenitor cells into osteoblasts such that the skeletal lesions of FD are composed largely of undifferentiated mesenchymal cells, which produce abnormal matrix, bone trabeculae, and collagen orientation.^2^^,^^7^^,^^9^ The timing of mutation in the developmental course determines the extent and type of FD, in which an earlier mutational event leads to a wider distribution of mutant cells, and consequently a more severe course of the disease.^1^^,^^2^

Fibrous dysplasia presents in monostotic and polyostotic forms. The more common monostotic form affects one bone and accounts for approximately 70% to 85% of cases. The polyostotic form affects several bones.^3^^,^^10^ In addition, the monostotic form manifests later in life, usually between 20 and 30 years of age,^2^ and most commonly affects the femur or the ribs.^3^ Majority of monostotic lesions are asymptomatic and found incidentally on radiographic imaging obtained for other indications.^1^ In contrast, the polyostotic form manifests earlier, usually in children younger than 10 years; has a more serious prognosis; and frequently affects the maxilla or other craniofacial bones, ribs, femur, or tibia.^2^ Three percent of polyostotic cases are associated with cafe-au-lait spots and a hyperfunctional endocrine state characteristic of McCune-Albright syndrome.^10^ This is more common in females than males (10:1) and more likely to cause precocious puberty in girls.^2^

Craniofacial FD refers to the involvement of multiple bones in the craniofacial skeleton, with 90% of these presenting before the age of 5 years.^7^ Although malignancy is rare (<1%), there is a risk of sarcomatous degeneration, which is increased by exposure to ionizing radiation.^2^^,^^7^ The diagnosis of FD is usually based on clinical, radiographic, and histopathologic features.^7^ Clinically, the most common presentation is swelling^3^^,^^10^; other manifestations include weakness, localized pain, deformity, fractures, and compromised vision or hearing.^1^ However, the majority are asymptomatic and discovered incidentally on radiographs as lesions with the characteristic ground glass appearance.

In such cases, a radiographic diagnosis is usually sufficient and a subsequent bone biopsy is not required. Lesions that pose minimal risks of pathologic fractures or deformities can be clinically observed depending on location, age of the patient, type of FD, and the patient's views toward surgery. Follow-up appointments should place special emphasis on the cranial nerves, with emphasis on careful ophthalmologic screening.^3^ A bone scan may be considered to rule out polyostotic FD, and follow-up radiographs recommended every 6 months to ensure there has been no progression.^1^ Computed tomography (CT) and magnetic resonance imaging are additional modalities for further elucidating the extent of bony and neurovascular involvement,^3^ and total body bone scintigraphy can determine the extent of skeletal disease and predict functional outcome.^1^^,^^7^

Appropriate treatment of FD is often highly individualized and based on patient-specific presentation.^11^ Lesions incidentally discovered by radiography can be conservatively managed. This typically involves bone scintigraphy to rule out polyostotic disease and serial clinical examinations of the lesion for any changes over time.^1^^,^^12^ When clinical evidence of changes over time have been noted, bisphosphonate therapy has shown promising results as the initial conservative treatment of FD.^12^ Observational studies report biphosphonates help improve function, decrease pain, and lower fracture risk.^1^^,^^3^^,^^7^ It is thought that elevated interleukin 6 may be responsible for the increased number of osteoclasts and subsequent bone resorption seen in FD, supporting the observation that pamidronate, a potent inhibitor of osteoclasts, can retard the spread of the disease into adjacent normal bone.^13^ Positive results have been reported with the use of pamidronate in relieving the pain associated with fibro-osseous lesions, normalization of bone turnover, and improvement of bone density. In cases refractory to medical management, definitive and long lasting results are obtained through surgical methods.^14^ Some of the indications for surgery following confirmatory biopsy include correction of unacceptable esthetic or functional deformity, prevention or correction of pathologic fracture, and removal of symptomatic lesions.^1^^,^^7^ Age at the time of presentation is another important consideration. Monostotic lesions tend to remain active only until skeletal maturity, whereas polyostotic lesions may progress during adulthood obviating the need for early surgical intervention in younger patients with monostotic disease.^1^ Although the majority of cases of monostotic FD are asymptomatic, indications for treatment include pain, new or progressive deformity, and disruption of function in the area of the lesion.^6^ Progressive or sudden visual loss is an absolute indication for surgical decompression,^15^^,^^16^ and high-dose corticosteroids can be initiated in conjunction with planning for rapid surgical intervention.^1^

Surgical intervention is aimed at preventing functional complications while improving regional esthetics.^14^ Surgical treatment ranges from curettage, bone remodeling, and contouring to more radical approaches such as total resection of involved bone with immediate reconstruction. Aggressive surgical techniques allow for complete removal of lesions and are the preferred method of treatment.^14^,^17^^-^19 Valentini et al^14^ in a retrospective study had no recurrences clinically or radiologically in 61 of 68 patients who underwent radical excision over a follow-up period of 7.6 years. Similarly, Maher et al reported on 48 patients with FD of the skull of which 7 patients had total resection and 17 underwent subtotal resection. After a mean clinical follow-up interval of 13.7 years, none of the patients who had total resections showed evidence of disease progression. The authors concluded that postoperative disease progression could be accurately predicted by the amount of resection as estimated by the surgeon or postoperative imaging. Resection of less than 90% of the dysplastic lesion was associated with disease recurrence.^18^ Although studies have shown that radical excision is the most effective treatment, there exists no universally accepted method of excision or grafting material for immediate reconstruction.

Chen and Noordhoff,^20^ and later Ricalde and Horswell,^3^ emphasized an approach to the treatment of craniomaxillofacial FD based upon classification of the skull into 4 zones and the relative reconstruction importance of each zone. Zone 1 includes the frontal, orbital, nasal, ethmoid, zygoma, and upper maxilla. The reconstruction importance of this zone is considered to be the fact that this area is the most conspicuous facial region and thus requires complete excision and reconstruction. Zone 2 includes the parietal, part of the occipital area and the lateral cranial base of the temporal zone; the reconstruction importance of this zone being hair-covered cranium that allows the option of conservative or radical resection. Zone 3 includes the central cranial base and petrous, mastoid, pterygoid, and sphenoid bones. This zone is difficult and oftentimes dangerous to manage, thus it is usually treated nonoperatively in the asymptomatic patient. Zone 4 is subdivided into 4a and 4b. The former includes the maxillary alveolar bone while the latter consists of the mandible. The reconstruction importance of this zone is its teeth-bearing feature, which allows for conservative excision and recontouring.

Murray et al^21^ employed 3-dimensional surgical imaging technology to first simulate and then perform complete excision reporting excellent cosmetic outcomes. The authors selected 3 patients with craniofacial FD that required extensive resection and used 3-dimensional imaging technology with haptic modeling to virtually simulate tumor excision and design of a polylactic-co-glycolic acid–implantable graft framework. The authors reported the resulting symmetric reconstruction was reliable and consistent resulting in a significant reduction in operative time as noted by other authors using similar techniques.^21^^-^24

## METHODS

**Figure 1:**
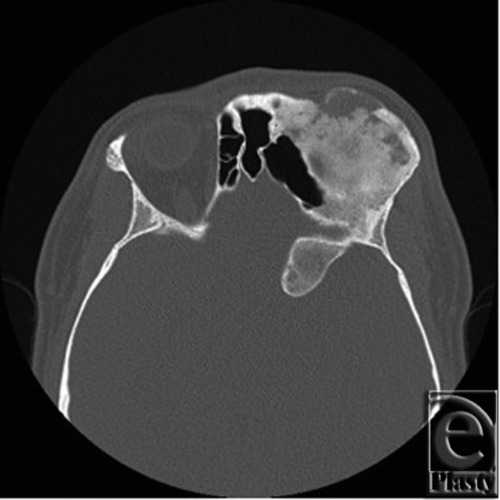
Preoperative axial computed tomographic image exhibiting characteristic ground-glass opacification of fibrous dysplasia involving the left frontal, temporal, and sphenoid bones.

**Figure 2:**
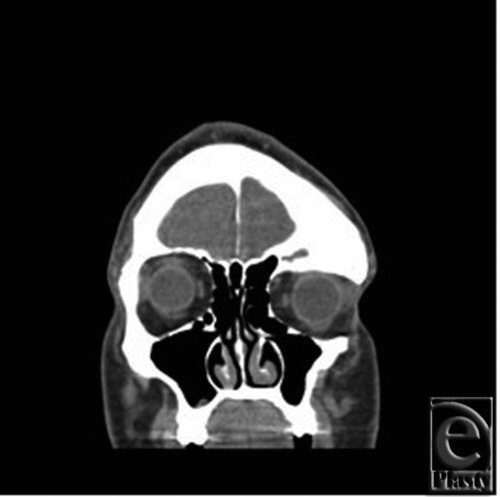
Preoperative coronal computed tomographic image demonstrating dysplastic growth of the left temporal bone with frontal involvement and displacement of orbital contents.

The patient is a 34-year-old, otherwise healthy, man who was diagnosed with FD at the age of 16. The patient was referred to the Plastic Surgery clinic after initial evaluation with the Neurology and Neurosurgical Services. He presented with complaints of change in quality and frequency of his headaches, occurring 2 to 3 times per week compared to his previous baseline of 2 to 3 times per month, as well as symptoms of blurry vision in the left eye. The patient also reported an increasing concern regarding the cosmetic appearance of the involved areas. Ophthalmologic evaluation confirmed decreased visual acuity in the left eye and optic nerve compromise. Computed tomographic imaging was obtained and these images are seen in Figures 1 and 2. Figure 1 demonstrates the characteristic ground glass opacification involving the left frontal, temporal, and sphenoid bones as well as involvement of the orbit with resulting vertical dystopia demonstrated in Figure 2. The thickness of the involved areas can also be appreciated in comparison to the contralateral orbit with bony expansion along the left anterior clinoid and the walls of the optic canal.

## RESULTS

**Figure 3:**
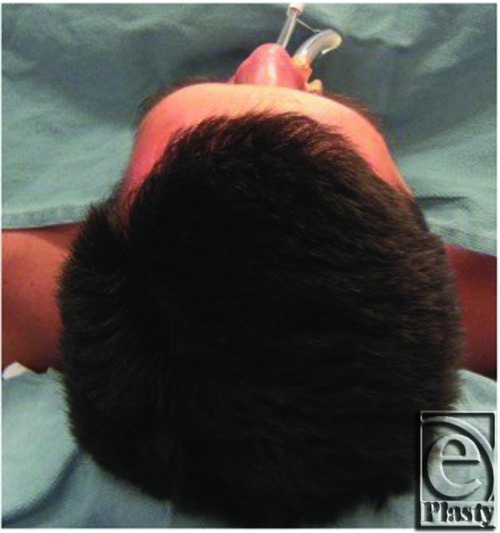
Intraoperative “bird's eye view” of the forehead highlighting the gross asymmetry of the left supraorbital rim in comparison to the contralateral side.

**Figure 4:**
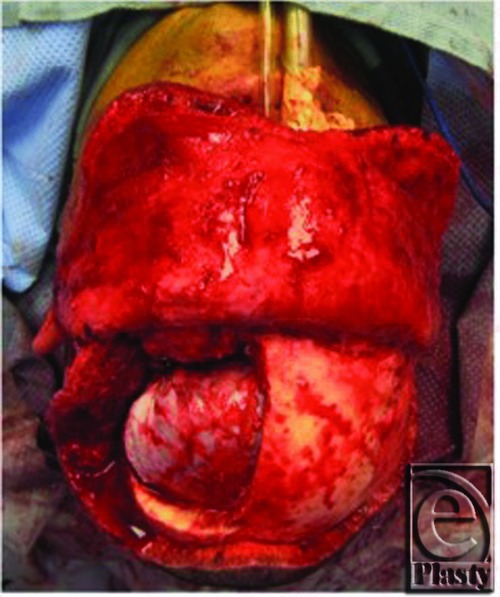
Intraoperative view following craniectomy.

**Figure 5:**
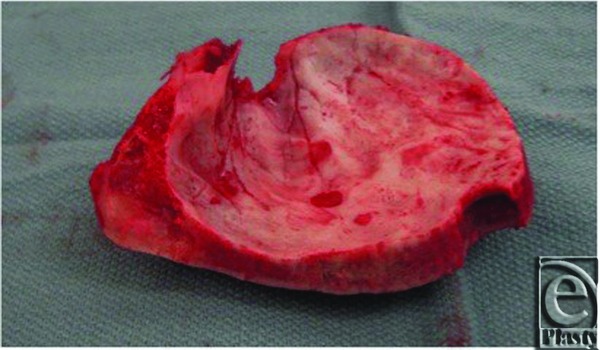
Postcraniectomy bone flap displaying extremely thickened abnormal bone.

**Figure 6:**
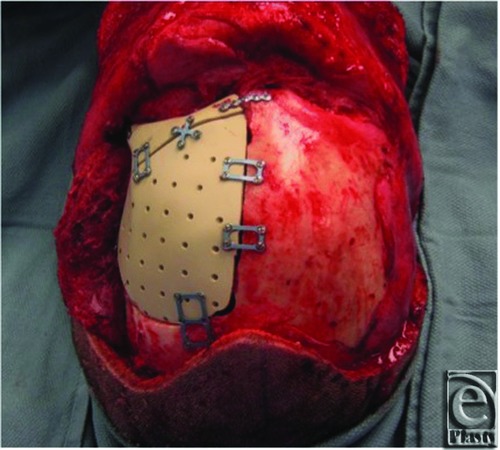
Intraoperative view following fixation of polyetheretherketone (PEEK) implant.

**Figure 7:**
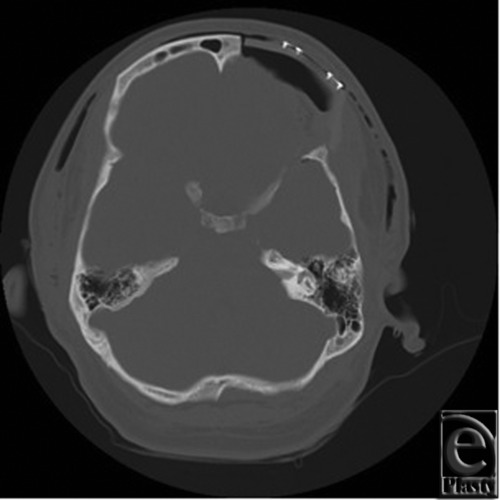
Postoperative computed tomographic scan showing polyetheretherketone implant in place at the frontal bone.

**Figure 8:**
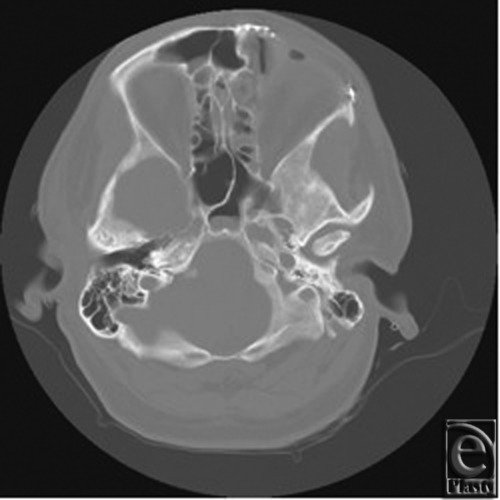
Postoperative computed tomographic scan showing supraorbital rim following reconstruction.

**Figure 9:**
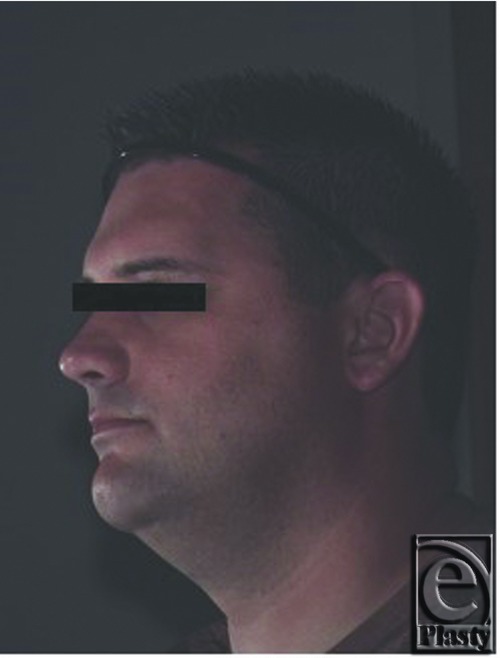
One-year follow-up demonstrating improved facial symmetry and supraorbital bar contour.

Following appropriate preoperative workup, the patient was taken to the operating room and a left fronto-temporo-orbitozygomatic craniotomy and cranioplasty as well as microsurgical decompression of the optic nerve was performed. In Figure 3, the prominence of the left supraorbital ridge is appreciated. Following a bicoronal incision and appropriate exposure, the craniotomy was performed and resulting defect following craniectomy is shown (Fig 4). Using the operating microscope, the orbital roof was unroofed, the anterior clinoid resected, and full decompression of the optic nerve was performed. The resected specimen is shown with the black arrow indicating the supraorbital rim (Fig 5). A Synthes Polyetheretherketone (PEEK) implant, which was prefabricated using the patient's preoperative CT scans, was used for alloplastic reconstruction. The predesigned implant was fashioned in 2 individual parts to allow for flexible and independent contouring of the forehead and the supraorbital ridge. The implants were contoured in the operating room for final fit. Figure 6 shows the PEEK implant in place and Figures 7 and 8 are the CT images taken after reconstruction. Four months later, the patient developed a cerebrospinal fluid leak for which a lumbar drain was initially placed followed by a lumboperitoneal shunt placement for recurrence. This resolved within the next month and the patient was doing well at the time of his 1-year follow-up. Figure 9 shows the patient at follow-up 1 year and 3 months later.

## DISCUSSION

First described by Von Recklinghausen in 1891, FD is an abnormal growth pattern characterized by fibro-osseous replacement of mature marrow with immature abnormal matrix. The abnormal production of fibroconnective tissue is due to a mutation in the *GNAS1* gene, which causes activation of a Gs protein leading to increased adenylate cyclase and upregulation of undifferentiated osteoprogenitor cells. Developmental timing of the mutation determines the severity of disease with earlier mutation leading to earlier manifestations and widespread disease. Two forms of FD exist, a monostotic and polyostotic form. Polyostotic disease tends to present at a younger age involving more than 1 bony area, whereas monostotic disease may remain nascent only manifesting itself radiologically, leading to the diagnosis of fibrous dysplasia. While the diagnosis of FD is not an absolute indication for treatment, symptoms resulting in functional impairment may warrant surgical intervention.

The management of FD involves the integration of multiple factors that add levels of complexity in determining the most appropriate treatment. In asymptomatic cases without evidence of disease progression, conservative management with serial examinations and imaging may be appropriate. In symptomatic cases, conservative treatment with pamidronate has been shown to be effective in reducing pain associated with lesions and slowing progression, but it does not provide definitive treatment. To achieve complete resolution of disease, surgical interventions provide the most reliable and long-lasting results.

## CONCLUSION

We present the case of a patient with craniofacial FD with threatened vision. The patient successfully underwent reconstruction with a custom implant and decompression of the optic nerve. The patient was doing well at follow-up over a year later and had good cosmetic results as seen in his contour profiles postoperatively. While conservative management may be appropriate in asymptomatic cases of FD, advances in modern surgical techniques and imaging technologies allow symptomatic cases to be treated reliably and efficiently with complete resection, restoring function and improving facial aesthetics.
